# Genome-Wide Identification and Colchicine-Responsive Expression Profiling of the Tubulins (TUA and TUB) Gene Family in *Phoebe bournei*

**DOI:** 10.3390/biology15171456

**Published:** 2026-08-26

**Authors:** Xiaowen Li, Jun Shen, Zeping Jian, Guoxin Mei, Bao Liu, Wenjun Lin, Shipin Chen, Haichao Hu

**Affiliations:** 1 College of Forestry, Fujian Agriculture and Forestry University, Fuzhou 350002, China; 12404021007@fafu.edu.cn (X.L.); jianzeping1015@163.com (Z.J.); 18291693423@163.com (G.M.); fafulb@163.com (B.L.); linwenjun123@126.com (W.L.); 2 Fujian Province Yunxiao County Forestry Bureau, Zhangzhou 363300, China; fjzzyxlyj@163.com

**Keywords:** *Phoebe bournei*, polyploid breeding, tubulin gene family, candidate gene, RNA-seq

## Abstract

*Phoebe bournei* is an economically and ecologically important woody species native to China. As a core component of colchicine-triggered polyploid breeding, the tubulin genes (TUA and TUB) have been identified and functionally analyzed in many plants, but not yet in *P. bournei*. Here, tubulin family members in *P. bournei* were identified through sequence alignment and subsequently characterized using comprehensive bioinformatic analyses. In particular, a total of six *PbTUA* and ten *PbTUB* members were identified and grouped into two and five subfamilies, respectively, according to phylogenetic relationships. Most tubulin proteins were small (414–522 aa) with predicted stability. Furthermore, 36 collinear gene pairs were identified, suggesting a possible contribution to the evolutionary expansion of this family. For different tissues, the expression levels of most tubulin genes were generally lower in leaves but higher in roots. Besides, treatment with 1.0% colchicine inhibited the expression of all 15 tubulin genes except *PbTUB6*. These results provide preliminary insights into tubulin genes associated with polyploid induction and supply candidate genes for future functional studies toward polyploid germplasm creation of *P. bournei*.

## Simple Summary

Tubulins assemble into mitotic spindles and play vital roles in polyploid formation. However, the tubulin gene family has not previously been characterized in *Phoebe bournei*, an economically and ecologically important native tree species. Here, we identified 16 tubulin genes and analyzed their evolutionary relationships, gene structures, conserved domains, cis-regulatory elements, tissue-specific as well as colchicine-induced expression patterns. Expression patterns across five tissues revealed that most tubulin transcripts accumulate preferentially in roots and stems. These genes may be involved in root development in *P. bournei*. Importantly, colchicine treatment induced pronounced downregulation in the expression of almost all tubulin genes. This work systematically characterizes the tubulin family in *P. bournei* and provides useful information for polyploid breeding.

## 1. Introduction

*Phoebe bournei* (Hemsl.) Yang belongs to evergreen woody plants within the genus *Phoebe* of the Lauraceae family. It is widely acknowledged as a high-value timber and ornamental tree native to southern China [1]. Owing to its distinctive fragrance, strong durability, favorable decay resistance, and appealing golden-colored wood, *P. bournei* has long been utilized for premium furniture and architectural applications throughout Asia [2]. Additionally, it plays critical roles in maintaining soil fertility and ecosystem stability [3]. However, its considerable commercial value has led to extensive human harvesting of *P. bournei*, resulting in serious depletion of its germplasm resources. High survival pressure and long growth cycles have restricted the development of production resources and also affected ecological restoration [4]. Therefore, developing polyploid breeding strategies and creating new germplasm resources are of vital importance to the sustainable advancement of the *P. bournei*-related industry.

Polyploids are organisms whose somatic cells possess at least three complete chromosome complements. For forest tree improvement, polyploidization can provide multiple beneficial traits, including enlarged vegetative organs, increased accumulation of metabolic products, and enhanced adaptability and stress resistance [5]. Tetraploid *Populus* seedlings exhibit increased stem diameter, leaf size, and stomatal size, indicating excellent growth potential [6]. Compared with its diploid counterpart, the tetraploid displayed thicker palisade tissue together with a lower stomatal density [7]. Autotetraploid soybeans showed enlarged tissues and elevated contents of oleic acid, water-soluble protein, and amino acids [8]. Tetraploid cumin showed 30–100% higher essential oil yield than diploids [9]. Triploid poplars could achieve a two- to three-fold increase in volumetric growth relative to diploid controls, potentially resulting in greater timber production and better wood properties [10]. Forest tree polyploids may originate spontaneously or be produced through artificial induction, with colchicine treatment being among the most commonly adopted methods for chromosome doubling. In *Cinnamomum*, a 24-h exposure of camphor tree seeds to 0.8% colchicine produced tetraploids at a frequency of 33.33% [11]. For the elite *A. melanoxylon* clone SR3, an in vitro regeneration system using bud-bearing stem segments achieved a polyploid induction frequency of 12.26 ± 0.80% when 5-day pre-cultured tissues were exposed to 100 mg/L colchicine for 72 h [12]. At present, successful colchicine-mediated polyploid induction has been reported in several plant species, such as *Populus canescens* [13], *Cyclocarya paliurus* [14], *Lycium chinense* [15], and *Aerides rosea* [16]. Previous research on woody-plant polyploids has mainly emphasized morphological characteristics [17], but the colchicine-induced transcriptional changes during polyploidization remain poorly documented across woody plant species. Colchicine induces chromosome doubling by specifically binding to tubulin heterodimers, thereby inhibiting microtubule polymerization and impairing mitotic spindle formation at metaphase [18]. Consequently, chromosome migration toward the spindle poles is suppressed, ultimately leading to whole-genome duplication in somatic cells.

Microtubules, indispensable core components of the plant cell cytoskeleton, are universally distributed in eukaryotes and predominantly consist of α-tubulin (TUA) and β-tubulin (TUB) [19]. TUA and TUB possess three structural segments: the N-terminal domain, intermediate domain and C-terminal domain. TUB carries inherent enzymatic capacity, with its N-terminal domain harboring the GTP-binding site. Once GTP attaches to this site, hydrolytic conversion to GDP drives reassembly of disassembled tubulin monomers, allowing microtubules to sustain dynamic switching between expansion and contraction states [20]. Meanwhile, plant cells exhibit a distinctive pattern of microtubule organization, in which microtubule nucleation can take place at multiple cortical and intracellular sites, such as the plasma membrane, the nuclear envelope, the surface of plastids, and undiscernible cytoplasmic MTOCs [21]. In plants, the dynamic turnover of microtubules contributes to morphogenesis, hormonal signaling, developmental processes, and responses to environmental stress [22]. Microtubule rearrangement and cell wall remodeling help plants adapt to environmental stresses. Transient *PaTUA1* overexpression was accompanied by short-term increases in the average levels of IAA, GA3, BR, and cytokinin within wounded developing apricot kernels [23]. Hybrid poplar seedlings respond to salinity stress through a mechanism involving the microtubule-associated protein PagPCaP1a [24]. During somatic cell division, numerous microtubules can be observed surrounding the nuclear envelope during prophase [25]. Perinuclear microtubules gradually organize into a spindle-like configuration. Upon nuclear envelope breakdown (NEBD), microtubules from perinuclear aggregates attach to kinetochores and assemble into a fully functional mitotic spindle. The metaphase spindle adopts a barrel-shaped architecture, with locally interconnected kinetochore-associated and non-kinetochore microtubules [26]. Upon the onset of anaphase, sister chromatids are separated and transported toward opposite cell poles via depolymerization of kinetochore-bound microtubules [27]. It is generally believed that inhibition of spindle assembly may promote the generation of polyploid cells. The kinetochore scaffold 1 (KNL1) functions as a central mediator linking kinetochores to the spindle assembly checkpoint (SAC), and recruits SAC-related proteins. Prior to anaphase onset, the SAC informs the cell cycle machinery of potential errors in the interactions between chromosomes and spindle microtubules, thus guaranteeing accurate chromosome segregation during mitosis. Extending from the inner kinetochore, the outer kinetochore can directly bind spindle microtubules [28,29]. When the spindle is perturbed, *Arabidopsis* delays mitosis in a SAC-dependent manner. Nevertheless, cells may still enter anaphase with duplicated chromosomes even in the absence of proper kinetochore-microtubule connections [30,31]. Under high-concentration treatment, the newly formed microtubule-containing structures can reorganize the 4C nucleus and drive entry into the cell cycle. Therefore, the mechanism by which colchicine induces chromosome doubling is closely associated with the structure and function of spindle microtubules. Tubulins undergo rapid remodeling and adopt dynamic spatial configurations, which strongly influence mitotic spindle assembly. Nevertheless, the transcriptional changes of tubulin genes following colchicine treatment remain unclear.

Tubulin gene families have been investigated in many agricultural and forestry species, including wheat [32], flax [33], upland cotton [34], and *Populus deltoides* [21]. Nevertheless, genome-wide characterization of the tubulin gene family in *P. bournei* remains unreported, and the expression patterns of *P. bournei* tubulin genes under colchicine treatment remain unclear. Hence, this study utilized the assembled *P. bournei* genome to identify the TUA and TUB gene families. Comprehensive analyses of chromosomal localization, phylogenetic relationships, protein architecture, gene organization, conserved motifs, cis-acting regulatory elements, and collinearity were carried out. Additionally, we investigated the dynamic expression profiles of *PbTUA* and *PbTUB* genes in different tissues and under colchicine treatments with varying durations using *P. bournei* seeds. This work was intended to establish a basis for future functional characterization of the *PbTUA* and *PbTUB* families in *P. bournei*.

## 2. Materials and Methods

### 2.1. Plant Material and Treatment

Mature seeds of *P. bournei* were collected from Jianxi Town, Nanping City, Fujian Province, China (117°32′44″ E, 26°30′50″ N) (Figure S1), followed by cold stratification to break seed dormancy and enhance germination. When the radicles began to emerge, the germinated seeds were then treated with a mixed solution of 1.0% colchicine (Shanghai Een Chemical Technology Co., Ltd., Shanghai, China) and 2% dimethyl sulfoxide (DMSO) (Aladdin Biochemical Technology Co., Ltd., Shanghai, China) for 24 h (TR10_24), 48 h (TR10_48), 72 h (TR10_72). Seeds from the control group were immersed in distilled water (MCK0). All treatments were conducted in darkness on a shaker incubator (HZQ-F160, Suzhou Peiying Experimental Equipment Co., Ltd., Suzhou, China) under 25 °C and set to an agitation rate of 50 r min^−1^. Thirty seeds were applied for each replicate, and three biological replicates were established for every experiment. After treatment, seeds were fully rinsed by distilled water to wash away residual colchicine and alleviate its phytotoxic damages, then immediately sown into 50-cell plug trays. The growing substrate consisted of peat, perlite and vermiculite mixed at a volume ratio of 6:3:1. The substrate was kept moist by watering every three days. Trays were incubated inside an illuminated growth incubator (TLST 01, Huaqi Technology Fujian Co., Ltd., Fuzhou, China). The environmental parameters were maintained at 25 ± 2 °C and 75 ± 5% relative humidity. LED lighting provided a photosynthetic photon flux density (PPFD) of 50 μmol·m^−2^·s^−1^ under a 16-h light / 8-h dark cycle. Fresh root samples were collected as experimental materials after 150 days of seedling growth. Root materials were harvested, flash-frozen with liquid nitrogen and preserved at −80 °C. The frozen samples were subsequently delivered to BGI Genomics Co., Ltd. Shenzhen, China, for transcriptome sequencing in January 2024. Total RNA was isolated from three *P. bournei* root samples with the PureLink™ Plant RNA Kit (Invitrogen, Carlsbad, CA, USA). To guarantee RNA purity and integrity, RNase Free DNase (Promega, Madison, WI, USA) was applied to remove any remaining DNA contamination. Subsequently, total RNA integrity was evaluated on a 1.25% agarose gel, whereas the purity of RNA samples was determined via a Nanodrop 1000 spectrophotometer (Thermo Scientific, Waltham, MA, USA). The resulting cDNA libraries were sequenced in paired-end (PE) mode using the Illumina HiSeq platform.

### 2.2. Identification and Chromosome Localization of TUA and TUB Gene Family Members in P. bournei

Reference gene sequences for *P. bournei* were retrieved from the China National GeneBank (https://ftp.cngb.org/pub/CNSA/data5/CNP0002030/CNS0395682/, accessed on 26 March 2026) to identify members of the TUA and TUB gene family. TUA and TUB protein sequences from *Arabidopsis thaliana* were retrieved from TAIR (https://www.arabidopsis.org/, accessed on 26 March 2026) and served as query sequences for identifying homologous TUA and TUB members in the *P. bournei* genome. A BLAST+ version 2.12.0 search was performed on the whole genome sequence of *P. bournei* using TBtools version 2.458 software to retrieve candidate protein sequences [35]. Candidate TUA and TUB members were retained using an E-value cutoff of 1 × 10^−5^. Conserved domains were verified with the NCBI (https://www.ncbi.nlm.nih.gov/Structure/cdd/wrpsb.cgi, accessed on 28 March 2026) Conserved Domain Database (CDD). Redundant, incomplete, and incorrectly annotated sequences were removed. Chromosomal positional information was extracted from General Feature Format (GFF) annotation files and visualized by TBtools v2.458. The confirmed TUA and TUB family members in *P. bournei* were renamed based on their chromosomal positions.

### 2.3. Physicochemical Property Analysis and 3D Model Construction of PbTUA and PbTUB Families

The amino-acid length, molecular mass, and theoretical isoelectric point of each identified *PbTUA* and *PbTUB* protein were estimated with the ExPASy proteomics server (https://www.expasy.org/, accessed on 24 April 2026). Subcellular localization predictions were implemented using the WoLF PSORT (https://wolfpsort.hgc.jp/, accessed on 25 April 2026). Structural templates were searched within the SWISS-MODEL template repository (https://swissmodel.expasy.org/, accessed on 28 April 2026). Suitable templates were selected based on their Global Model Quality Estimate (GMQE) values and sequence homology. For all *PbTUA* and *PbTUB* proteins, the chosen templates exhibited sequence identities greater than 80% and GMQE values above 0.80, supporting the reliability of the predicted three-dimensional models.

### 2.4. Phylogenetic Analysis of PbTUA and PbTUB

TUA and TUB protein sequences were aligned with the ClustalW algorithm implemented in MEGA12 (Table S1) [36]. The resulting alignments were used to reconstruct a phylogenetic tree in MEGA12 using the Neighbor-Joining method, with 1000 bootstrap replicates. All remaining parameters remained set to default values. The resulting tree was graphically presented using iTOL (https://itol.embl.de/, accessed on 19 May 2026).

### 2.5. Analysis of Conserved Motifs, Cis-Regulatory Elements, and Gene Organization of PbTUA and PbTUB

Prediction of conserved motifs across the protein sequences was performed via the MEME Suite (https://meme-suite.org/meme/tools/meme, accessed on 30 April 2026). The number of motifs was specified as 10, whereas the remaining settings were left unchanged. The exon–intron structures of the *PbTUA* and *PbTUB* genes were displayed according to alignments between genomic and coding sequences, extracting relevant information from the GFF annotation file. Then, TBtools v2.458 was applied to retrieve 2000-bp upstream DNA fragments of *PbTUA* and *PbTUB* from the *P. bournei* genome assembly, which were regarded as promoter regions for downstream analysis. The PlantCARE database (https://bioinformatics.psb.ugent.be/webtools/plantcare/html/, accessed on 9 April 2026) was utilized to screen cis-regulatory elements harbored within *PbTUA* and *PbTUB* gene promoters.

### 2.6. Synteny Analysis of PbTUA and PbTUB Families

Genome data for *Populus trichocarpa* were obtained from the Ensembl Plants website (https://plants.ensembl.org/index.html, accessed on 24 April 2026). Gene sequences together with annotation documents from *P. bournei*, *A. thaliana* and *P. trichocarpa* were imported into the “One Step MCScanX” module of TBtools v2.458 to conduct collinearity assessment. Cross-species syntenic relationships were displayed using the Dual Synteny Plot function. Intra-genomic collinearity of the *P. bournei* genome was analyzed with “One Step MCScanX”, and intraspecific synteny was presented with “Advanced Circos”. For the analysis, the BLASTP version 2.12.0 CPU parameter was assigned a value of 2, while the E-value cutoff was fixed at 1 × 10^−10^. Synonymous substitution rates (Ks), nonsynonymous substitution rates (Ka), and their Ka/Ks ratios were estimated with the Simple Ka/Ks Calculator (NG) in TBtools v2.458. Ka/Ks values below 1, equal to 1, and above 1 were interpreted as evidence of purifying, neutral, and positive selection, respectively.

### 2.7. RNA-Seq Data Analysis and Expression Analysis of PbTUA and PbTUB Families

Transcriptome sequencing data of different *P. bournei* tissues were retrieved from NCBI, with the BioProject accession number PRJNA628065. Transcriptome sequencing was performed on untreated (MCK0), 1.0% colchicine-treated for 24 h (TR10_24), 48 h (TR10_48), and 72 h (TR10_72), with the BioProject accession number PRJNA1496853. Transcriptomic reads were aligned to the chromosome-scale reference genome of *P. bournei*. SOAPnuke v1.5.2 was initially used to filter the raw reads and obtain high-quality clean sequences. Filtered reads were subsequently aligned to the *P. bournei* reference genome with HISAT v2.1.0 to assess data reliability. In addition, Bowtie2 v2.2.5 was applied to align clean data against reference gene sequences, and the RSEM package v1.2.8 was utilized to calculate gene expression levels for each sample, providing fundamental datasets for subsequent bioinformatic analyses. Following quality filtering, each sample generated 6.63–6.64 Gb of clean bases. Across all samples, Q20 values were greater than 98.18%, whereas Q30 values varied between 93.73% and 94.27%, with clean-read ratios higher than 98.05%. Clean reads were aligned against the *P. bournei* reference genome. The overall mapping rate fell within 91.83–93.99%, whereas the uniquely mapped read proportion fluctuated between 87.21% and 89.33%. Together, these quality metrics demonstrated that the RNA-seq datasets were sufficiently reliable for downstream bioinformatic analyses. Fragments Per Kilobase of transcript per Million mapped reads (FPKM) values of *PbTUA* and *PbTUB* family genes were extracted (Table S2). Expression profiles were visualized as a clustered heatmap in TBtools v2.458 after transformation of the values to log_2_(FPKM + 1). Based on the annotation results from GO and KEGG, the differential genes between MCK0 and TR10_24 were assigned to functional categories. Functional enrichment for GO terms and KEGG pathways was evaluated with the phyper function in R. To define the criteria for significant enrichment, a threshold of Q-value ≤ 0.05 was set.

### 2.8. Protein–Protein Interaction Network of the PbTUA and PbTUB Families

A protein–protein interaction (PPI) network for *PbTUA* and *PbTUB* proteins was generated by submitting all protein sequences to STRING 12.0 (https://cn.string-db.org, accessed on 6 May 2026). Homology-based mapping was carried out with *A. thaliana* serving as reference species; the interaction confidence score was fixed at 0.7, and disconnected network nodes were concealed. The remaining settings were left unchanged.

## 3. Results

### 3.1. Identification and Chromosome Distribution of TUA and TUB Gene Families in P. bournei

**Figure 1:**
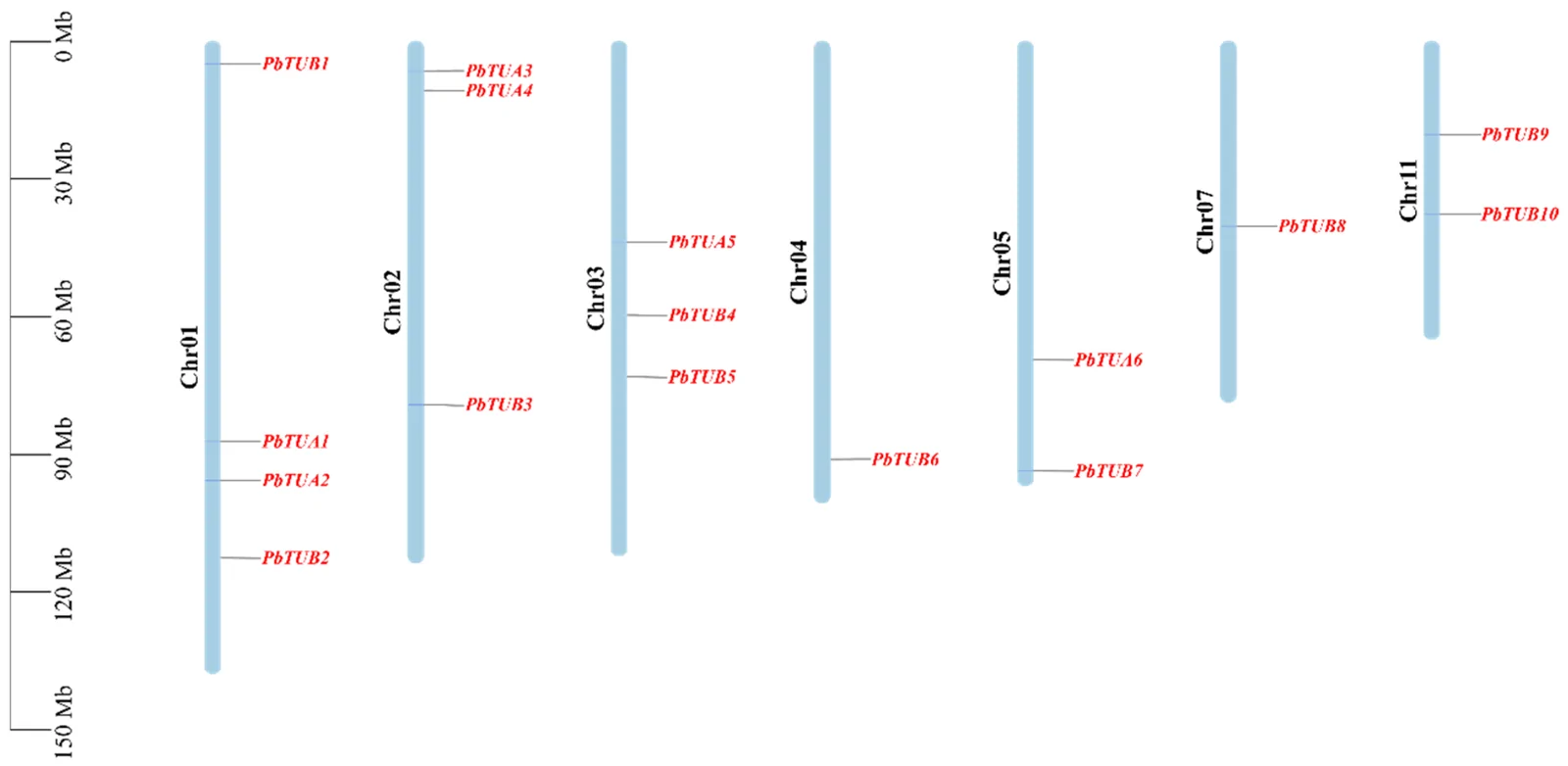
Chromosomal localization of *PbTUA* and *PbTUB* genes on *P. bournei* chromosomes. Blue bars represent individual chromosomes; red labels indicate the positions of *PbTUA* and *PbTUB* genes. Chromosome identifiers were shown above the bars, while the left-hand scale represented chromosome length in megabases (Mb). Relative chromosome lengths were drawn according to positional information obtained from GFF.

A total of 16 tubulin genes were detected in the *P. bournei* genome (Figure 1). All genes were located alone or in clusters on 7 chromosomes, and were designated according to their physical positions on the *P. bournei* chromosomes (*PbTUA1* to *PbTUA6* and *PbTUB1* to *PbTUB10*). The number of *PbTUA* and *PbTUB* members varied among chromosomes. Chr01 harbored the largest number of tubulin genes, with four members, whereas Chr03 and Chr02 each contained three. In contrast, Chr05 and Chr11 each carried only two tubulin genes. Less than 2 genes were found in Chr04 (1) and Chr07 (1). The uneven distribution of *PbTUA* and *PbTUB* on the chromosomes may imply distinct expression patterns, which might explain the absence of several conserved motifs.

### 3.2. Physicochemical Characteristics and Predicted Subcellular Localization of PbTUA and PbTUB Proteins

**Figure 2:**
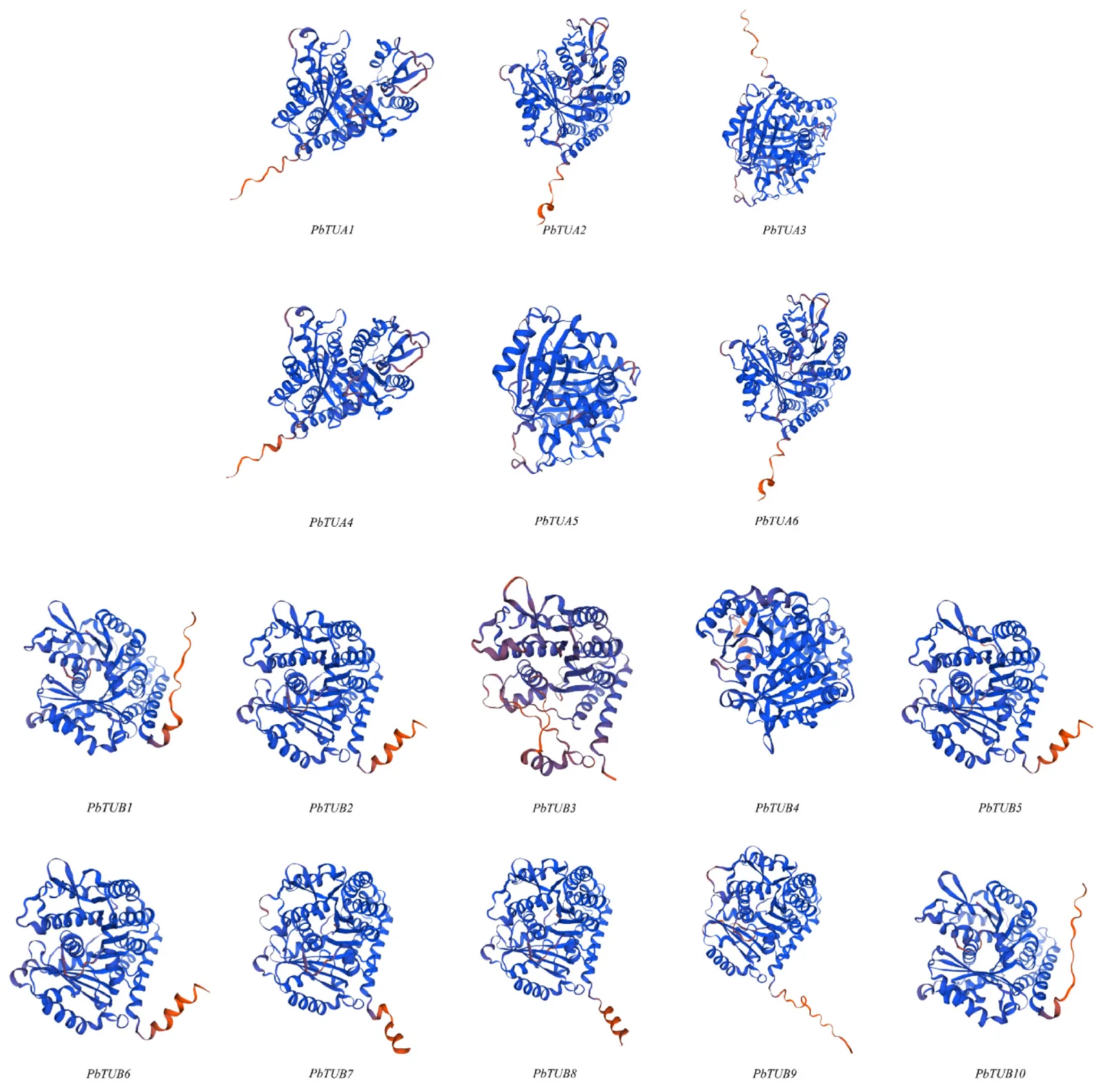
Predicted tertiary structures of *PbTUA* and *PbTUB* in *P. bournei*. The main protein-fold domains are shown in blue, and the C-terminal acidic tails are highlighted in orange.

Physicochemical properties, predicted subcellular localization, and tertiary protein structure prediction were conducted on members of the *PbTUA* and *PbTUB* gene families (Table 1 and Figure 2). The tertiary structures of TUA and TUB are highly similar, whereas their structural divergence originates from the C-terminal region [37]. Analysis showed that *PbTUA* genes encode proteins with lengths spanning 414 aa (*PbTUA1*) to 451 aa (*PbTUA2*, *PbTUA3*, and *PbTUA6*). Compared with *PbTUA* genes, *PbTUB* genes encode proteins with amino acids ranging from 430 aa (*PbTUB5*) to 522 aa *(PbTUB3*), with a slightly larger size span. The predicted molecular masses of *PbTUA* proteins varied between 45,809.80 and 49,771.31 Da, whereas those of *PbTUB* proteins ranged from 48,269.44 to 58,841.70 Da. The theoretical isoelectric point (pI) spanned 4.7 (*PbTUB9*) to 5.01 (*PbTUA1*), revealing that all 16 proteins were acidic with pI values below 7. Based on the instability index, only *PbTUB3* and *PbTUB8* had values above 40 and were therefore predicted to be unstable proteins. Negative grand average of hydropathicity (GRAVY) values were obtained for all proteins, demonstrating the hydrophilic nature of the 16 tubulin proteins. As for TUA members, *PbTUA1* was predicted to localize to the cytoskeleton, whereas the others were predicted to localize to the cytoplasm. Differently, *PbTUB* members were localized in the nucleus, except *PbTUB6* located in the cytoplasm.

### 3.3. Phylogenetic Analysis of PbTUA and PbTUB Gene Family Members in P. bournei

**Figure 3:**
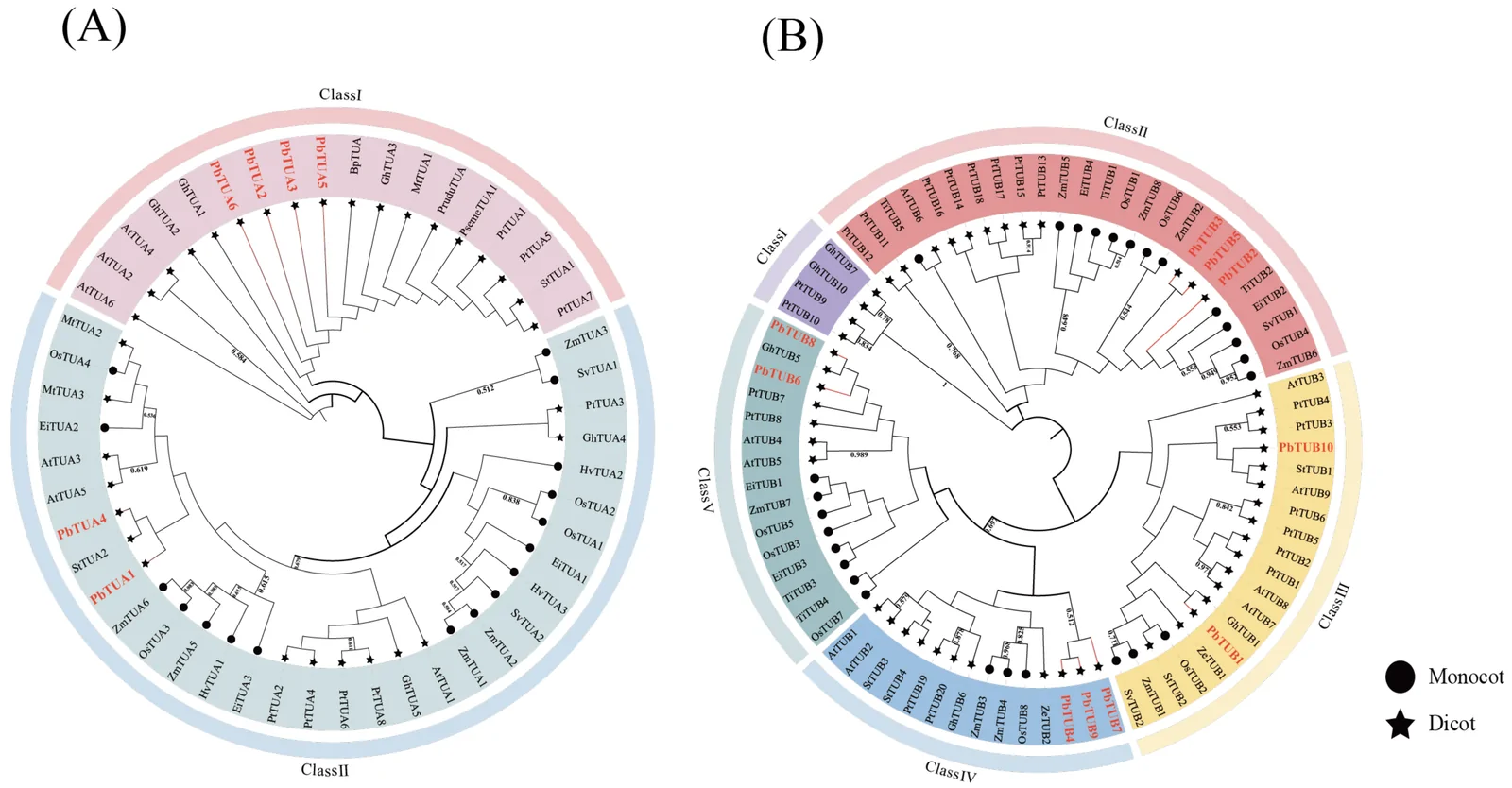
Phylogenetic tree of the tubulin gene family. (**A**) Phylogeny of TUA proteins. (**B**) Phylogeny of TUB proteins. At, *Arabidopsis thaliana*; Bp, *Betula pendula*; Ei, *Eleusine indica*; Gh, *Gossypium hirsutum*; Hv, *Hordeum vulgare*; Mt, *Medicago truncatula*; Os, *Oryza sativa*; Prudu, *Prunus dulcis*; Pseme, *Pseudotsuga menziesii*; Pt, *Populus trichocarpa*; St, *Solanum tuberosum*; Sv, *Setaria viridis*; Ti, *Triticum aestivum*; Zm, *Zea mays*; Ze, *Zinnia elegans*.

Phylogenetic relationships were reconstructed based on 50 TUA and 77 TUB proteins from 16 plant species (Figure 3). The phylogenetic results separated the TUA family into two principal clades, designated Class I and Class II (Figure 3A). The *PbTUA* form the majority of Class I, including *PbTUA2*, *PbTUA3*, *PbTUA5*, and *PbTUA6*; this branch exclusively contains TUA genes from dicot species. The Class II clade contains sequences from both monocot and dicot species. These results indicated that different *PbTUA* clades had undergone distinct levels of evolutionary divergence. The observed clustering pattern further suggests that the TUA family originated before the separation of monocotyledonous and dicotyledonous plants and has retained a relatively high degree of sequence conservation during subsequent evolution.

Depending on the species analyzed and the algorithms used, members of TUB gene family can be roughly divided into four to five main groups [38]. The 77 TUB protein sequences were grouped into five phylogenetic classes (Class I–V) (Figure 3B). *PbTUB* members showed a relatively balanced distribution among the five groups. The Class I clade exclusively comprised dicotyledonous species, whereas all other clades included both monocots and dicots, indicating that the TUB family may undergo an early evolutionary divergence within angiosperms. *PbTUB2*, *PbTUB3*, *PbTUB5* were grouped within Class II, *PbTUB1*, *PbTUB10* were assigned to Class III, *PbTUB4*, *PbTUB7*, *PbTUB9* belonged to Class IV, and *PbTUB6*, *PbTUB8* belonged to Class V. Genes assigned to the same phylogenetic group were likely to exhibit comparable gene organization, protein structure, and functional domains.

### 3.4. Conserved Motifs, Cis-Acting Elements, and Gene Structure

**Figure 4:**
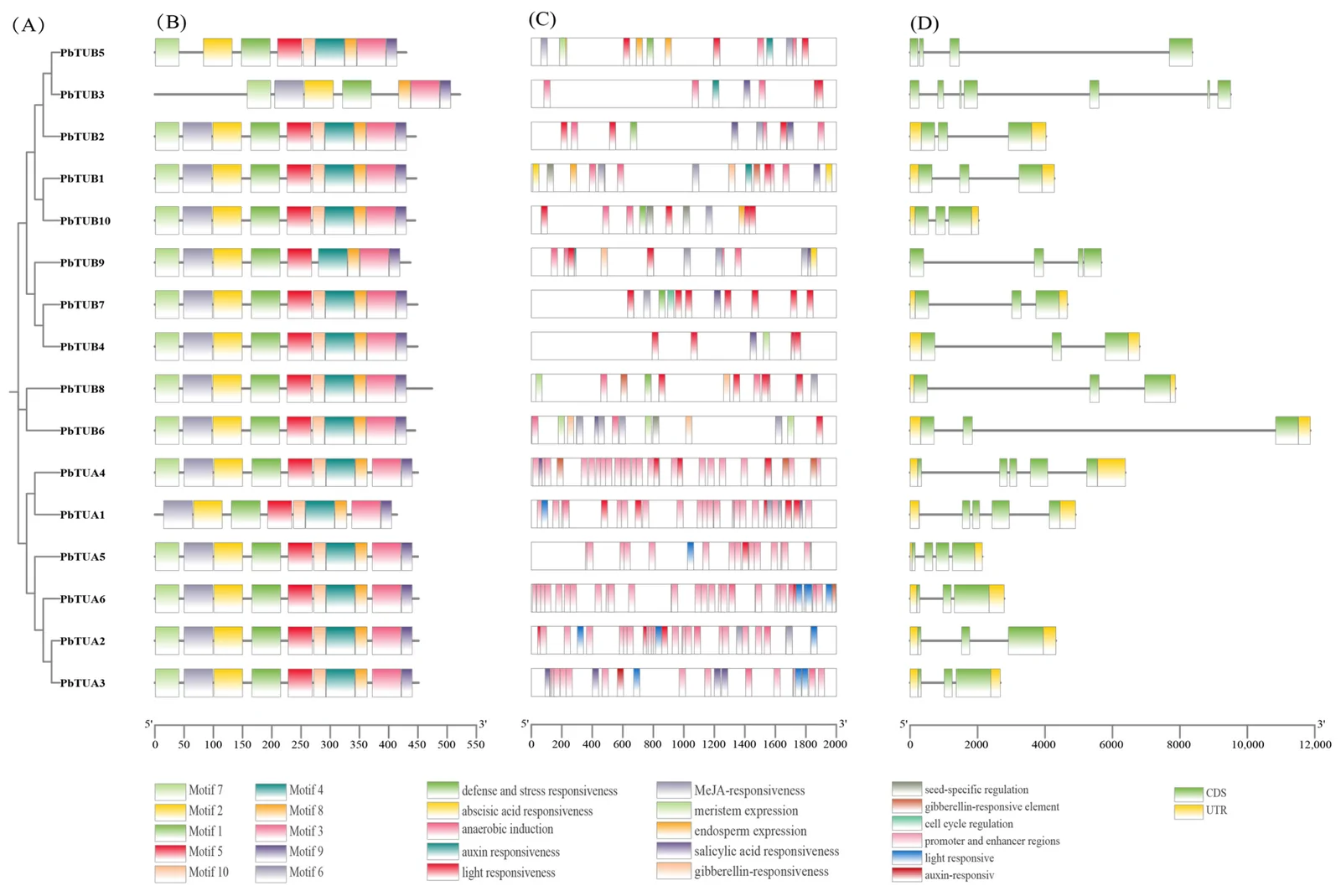
Analysis of *PbTUA* and *PbTUB* family characteristics. (**A**) Phylogenetic tree. (**B**) Conserved motifs. (**C**) Cis-acting element. (**D**) Gene structure.

Ten conserved motifs (motif 1-motif 10) were detected among *PbTUA* and *PbTUB* proteins (Figure 4B). Several motifs were missing in *PbTUA1*, *PbTUB3*, *PbTUB5* and *PbTUB9*, a phenomenon possibly caused by sequence divergence. As expected, closely related *PbTUA* paralogs grouped within the same phylogenetic clade displayed highly similar motif compositions, indicating conserved structural characteristics within the same subclade. Most *PbTUA* had nine motifs except motif 7; the *PbTUB* family contained the other seven motifs except motifs 4, 6, and 9. Specifically, *PbTUA1* lacked motif 7; *PbTUB3* was missing motif 9 and motif 4; *PbTUB5* lacked motif 6; and *PbTUB9* lacked motif 9. This suggested that the remaining motifs were critical for the function of the *PbTUA* and *PbTUB* families, and might have cooperatively participated in regulating the expression of specific traits.

Cis-acting regulatory elements serve as binding sites for transcription factors and exert core functions in modulating gene transcriptional activity. The 2000-bp regions upstream of the *PbTUA* and *PbTUB* genes were retrieved for promoter-element analysis, and the positional distribution of the predicted elements was subsequently illustrated (Figure 4C). In this study, the identified cis-acting elements were mainly divided into nine categories, including auxin response elements, light response elements, methyl jasmonate (MeJA) response elements, abscisic acid (ABA) response elements, gibberellin response elements, salicylic acid (SA) response elements, stress response elements, tissue-specific elements (meristematic elements, endosperm expression elements, seed-specific regulatory elements), and cell cycle regulatory elements. Among these, light responsive elements constituted the largest group (26%). They were followed by MeJA response (17%) and ABA response elements (16%). Additionally, the promoters harbored elements responsive to meristematic expression, endosperm-specific expression, seed-specific regulation, and cell cycle regulation. These results indicated that transcription of *PbTUA* and *PbTUB* might be jointly influenced by light signals, multiple phytohormones, and environmental cues.

The gene structures of *PbTUA* and *PbTUB* family members were highly similar, with exon numbers varying from 3 to 7 and intron counts varying between 2 and 6 (Figure 4D). Regarding untranslated regions, most genes possessed both 5′ and 3′ UTRs, while several members showed partial or missing UTR annotations (e.g., *PbTUB3*, *PbTUB5*, and *PbTUB9*). *PbTUB3* had seven exons and six introns, *PbTUA4* had five exons and four introns, the four members (*PbTUA1*, *PbTUA5*, *PbTUB5,* and *PbTUB9*) exhibited four exons and three to four introns, and the ten members containing only three exons and two introns. Overall, the similarity in *PbTUA* and *PbTUB* gene structures suggested that members of *P. bournei* were evolutionarily conserved.

### 3.5. Gene Replication and Collinearity Analysis

**Figure 5:**
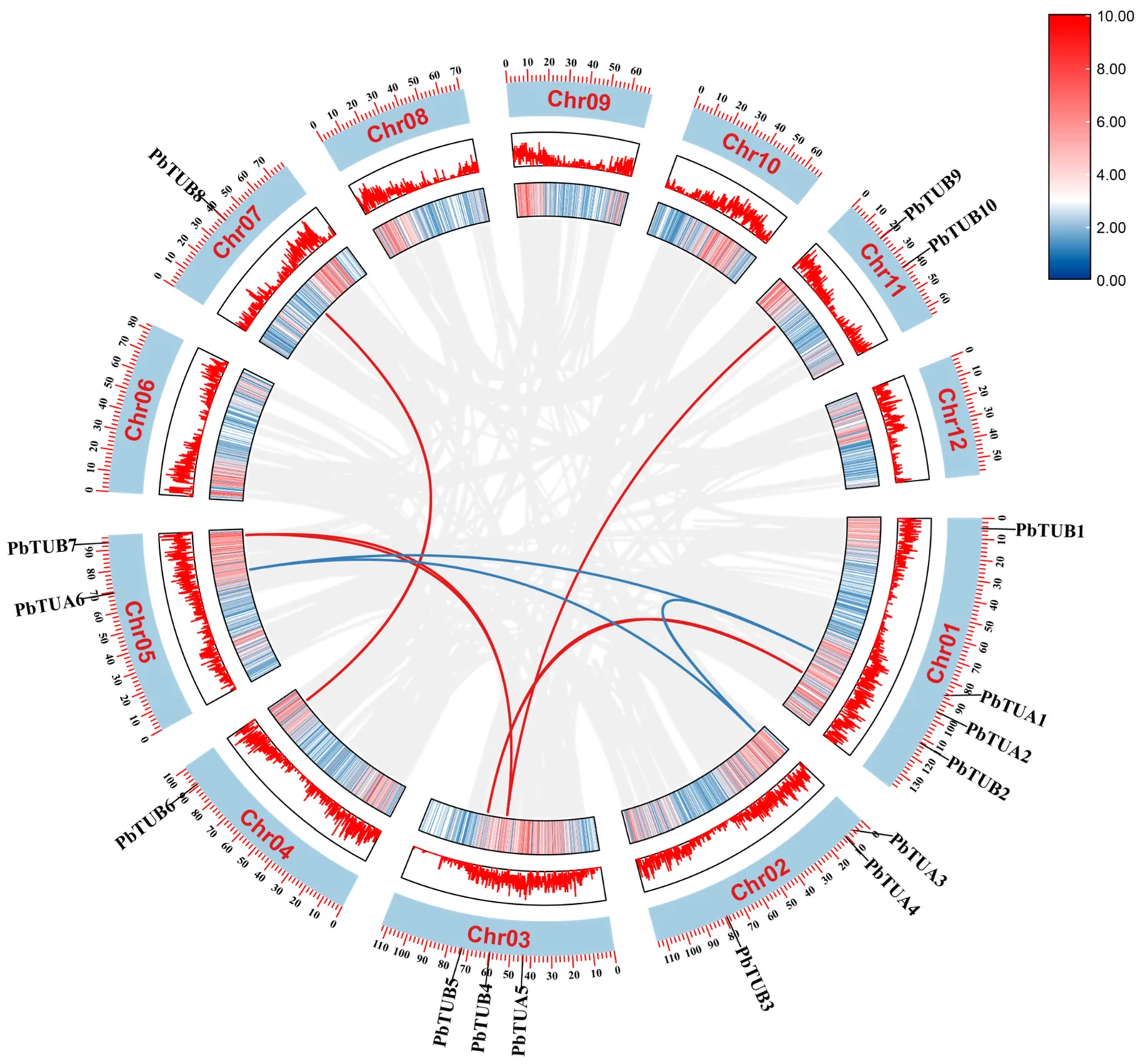
Collinearity analysis of *PbTUA* and *PbTUB*. Gray lines represented homologous genomic regions within *P. bournei*; segmentally duplicated *PbTUA* pairs were linked by blue lines, and red lines indicated collinear *PbTUB* gene pairs arising from segmental duplication.

Gene collinearity analysis could identify homologous sequences within species, and duplicated genes presented one of the processes that lay the foundation for the emergence of new genes. Several processes have been suggested to contribute to gene duplication, including whole-genome, tandem, proximal, transposed, and dispersed duplication events [39]. To determine whether duplication contributed to the evolution of *PbTUA* and *PbTUB* genes in *P. bournei*, segmental duplication patterns were examined. Seven collinear gene pairs located on different chromosomes were detected (Figure 5), providing evidence for segmental duplication within these families. Moreover, three segmentally duplicated pairs were detected among *PbTUA* members: *PbTUA2* with *PbTUA3*, *PbTUA2* with *PbTUA6*, and *PbTUA3* with *PbTUA6*. In the *PbTUB* gene family, there were four pairs of segmental duplications: *PbTUB2* with *PbTUB5*, *PbTUB4* with *PbTUB7*, *PbTUB4* with *PbTUB9*, and *PbTUB6* with *PbTUB8*. The Ka/Ks value of 0.02245129 (<1) suggested strong purifying selection for this gene pair (Table S3). These observations suggested that segmental duplication acted as a major driving force for the expansion of *PbTUA* and *PbTUB* gene families in *P. bournei*.

**Figure 6:**
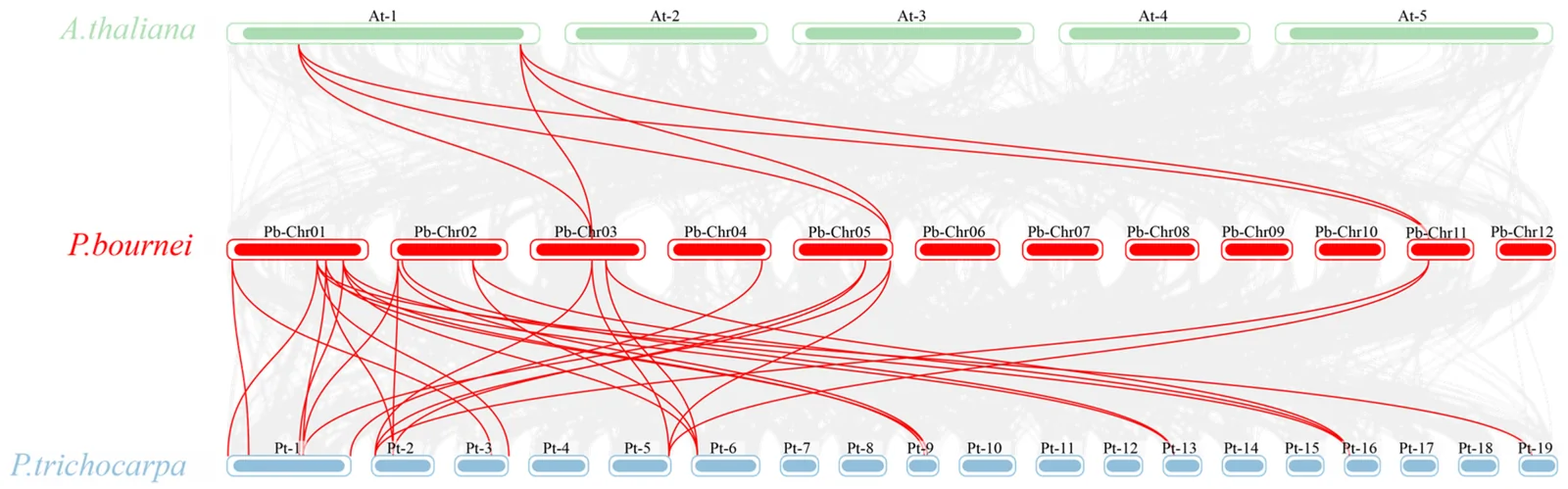
Syntenic relationships between TUA and TUB genes in *P. bournei* with other TUA and TUB genes in three other representative plant species. Background gray lines represent conserved collinear regions between the *P. bournei* genome and the genomes of the comparison species. Syntenic TUA and TUB pairs are connected by red lines.

To further examine the evolutionary relationships of the *PbTUA* and *PbTUB* families, interspecific synteny was analyzed. This analysis compared *P. bournei* with *A. thaliana*, and *P. trichocarpa* (Figure 6). The results revealed that *P. bournei* shared only six collinear tubulin gene pairs with the *A. thaliana* genome, while it shared 30 with the *P. trichocarpa* genome. The greater number of shared collinear pairs suggested a comparatively close evolutionary relationship between *P. bournei* and *P. trichocarpa*.

### 3.6. Expression Patterns of PbTUA and PbTUB Genes

**Figure 7:**
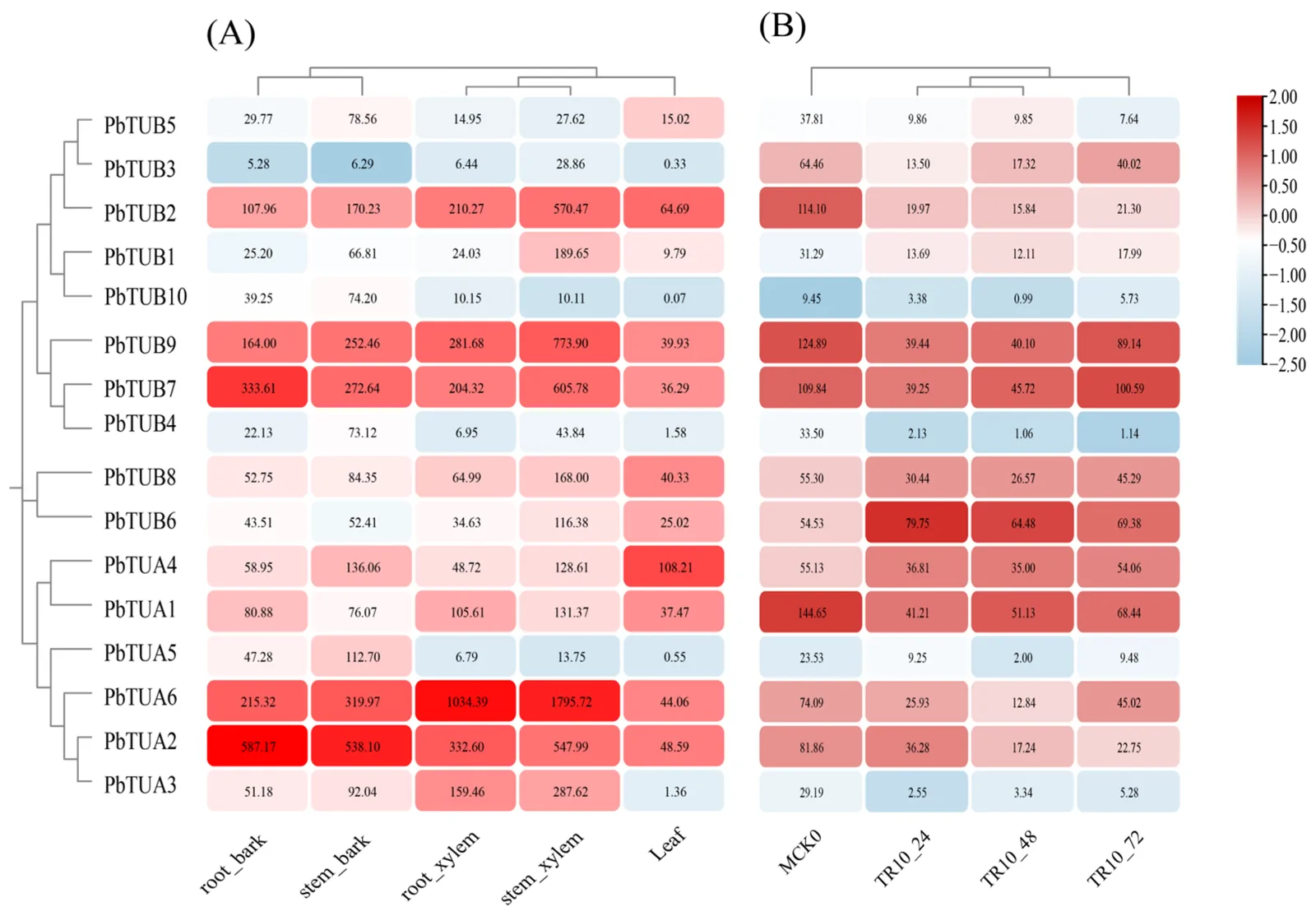
Expression patterns of *PbTUA* and *PbTUB* family. (**A**) Expression of *PbTUA* and *PbTUB* in five tissues. (**B**) Expression of *PbTUA* and *PbTUB* in MCK0, TR10_24, TR10_48, and TR10_72.

Gene expression profiles could offer valuable hints for deducing putative gene functions. To explore the possible roles of *PbTUA* and *PbTUB* genes during *P. bournei* growth and development, their expression levels were compared across five tissues: root bark, root xylem, stem bark, stem xylem, and leaves (Figure 7A). The results showed distinct expression profiles across different tissues. Specifically, *PbTUB4*, *PbTUB5*, *PbTUB10*, *PbTUA4*, *PbTUA5,* and *PbTUA6* were significantly expressed in roots; *PbTUB1*, *PbTUB2*, *PbTUB3*, *PbTUB6*, *PbTUB7*, *PbTUB8*, *PbTUB9*, *PbTUA1*, *PbTUA2*, and *PbTUA3* were expressed in stem tissues. Notably, *PbTUA4* exhibited an expression level of 108.21 (FPKM) in leaf tissues, which was markedly higher than other *PbTUA* and *PbTUB* members in the same tissue. The preferential expression of these *PbTUA* and *PbTUB* genes in specific tissues suggested that they might participate in root and stem development or related physiological functions, although experimental confirmation was still needed.

To explore the influences of colchicine exposure across different time points on expression profiles of *PbTUA* and *PbTUB* genes in *P. bournei*, a comparative analysis of gene expression levels in MCK0, TR10_24, TR10_48, and TR10_72 was conducted (Figure 7B). Under colchicine stress of varying exposure time, transcript abundance for the majority of *PbTUA* and *PbTUB* genes declined relative to the MCK0 control. Compared with the 24 h and 48 h treatments, the expression levels of *PbTUA4*, *PbTUA6*, *PbTUB3*, *PbTUB6*, *PbTUB7*, *PbTUB8*, *PbTUB9*, and *PbTUB10* displayed varying degrees of upregulation at 72 h. With increasing duration of colchicine treatment, *PbTUA* genes exhibited two distinct expression trends, while *PbTUB* genes displayed three major expression patterns. Among them, *PbTUA2*, *PbTUA4*, *PbTUA5*, and *PbTUA6* followed a “decrease-decrease-increase” expression profile, whereas *PbTUA1* and *PbTUA3* showed a “decrease-increase-increase” trend. For the *PbTUB* genes, *PbTUB1*, *PbTUB2*, *PbTUB4*, *PbTUB8*, and *PbTUB10* conformed to the “decrease-decrease-increase” pattern; *PbTUB3*, *PbTUB6*, *PbTUB7*, and *PbTUB9* exhibited a “decrease-increase-increase” trend, and *PbTUB5* displayed an overall declining expression tendency. In addition, *PbTUB6* exhibited consistently higher expression under all treatments than the MCK0 control group.

**Figure 8:**
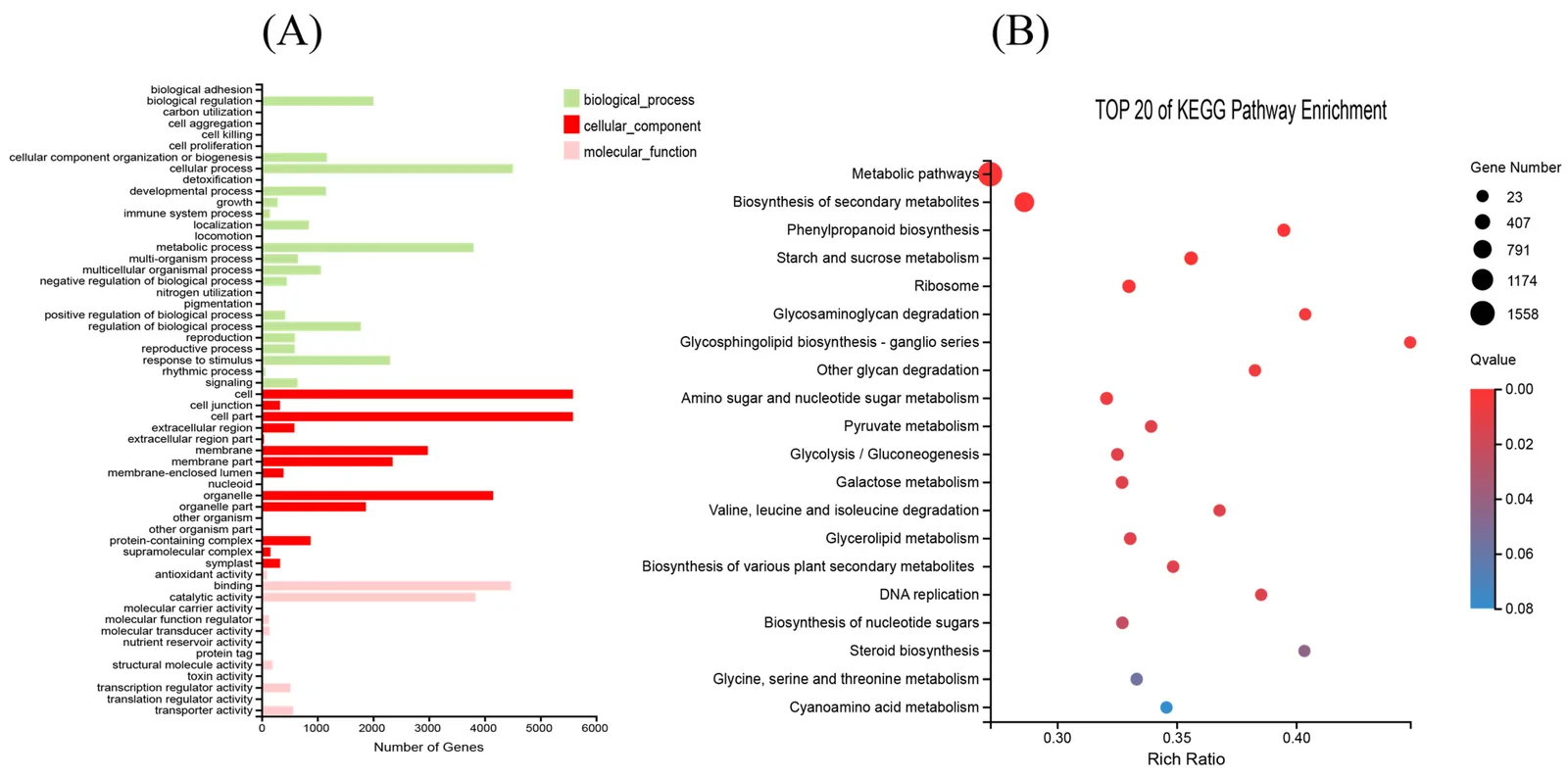
Functional enrichment analysis of DEGs between MCK0 and TR10_24. (**A**) GO classification. (**B**) KEGG enrichment map.

GO (Gene Ontology) and KEGG (Kyoto Encyclopedia of Genes and Genomes) pathway enrichment analyses were performed for differentially expressed genes (DEGs) from the MCK0 versus TR10_24 treatment comparison (Figure 8). GO annotation classified the identified DEGs into three principal functional categories: biological process (BP), molecular function (MF), and cellular component (CC). Within the CC category, most DEGs were enriched in cell and cell part terms, whereas the dominant BP terms included metabolic process and cellular process, and the most abundant MF terms were binding and catalytic activity, indicating that the DEGs were mainly involved in cellular structures, fundamental biological processes, and diverse biochemical activities. KEGG enrichment analysis identified metabolic pathways and biosynthesis of secondary metabolites as the most prominent pathways (Table S4). Multiple carbohydrate- and energy-associated pathways, such as starch and sucrose metabolism, gluconeogenesis, pyruvate metabolism, as well as amino sugar and nucleotide sugar metabolism, were likewise enriched.

### 3.7. Protein–Protein Interaction Network Analysis of the PbTUA and PbTUB Families

**Figure 9:**
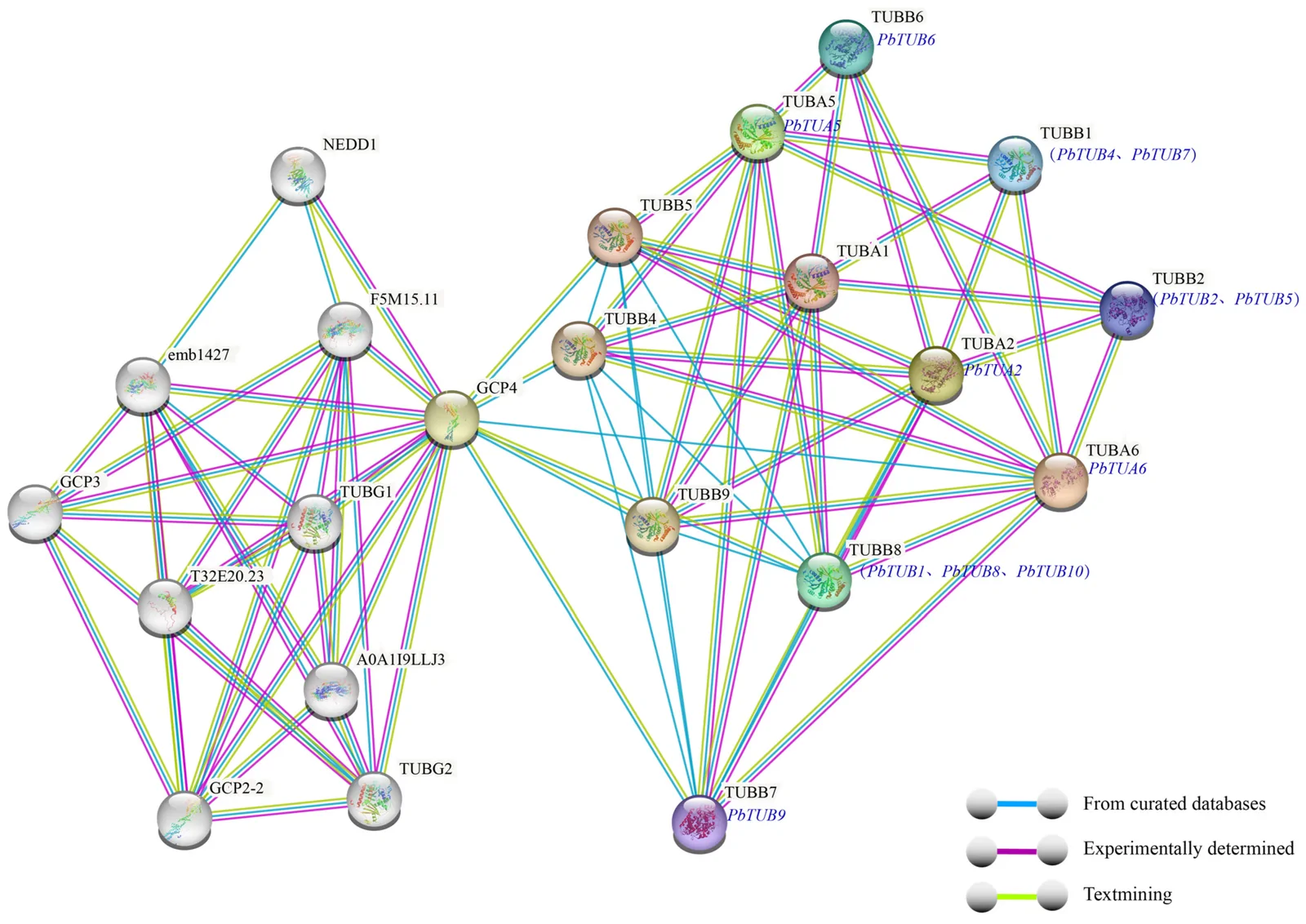
Predicted functional association network of *PbTUA* and *PbTUB*.

To comprehensively characterize interactions among *PbTUA* and *PbTUB* in *P. bournei*, a protein–protein interaction (PPI) network was established (Figure 9). Network topology identified *PbTUA2* and *PbTUA6* as the principal hub proteins. These proteins displayed substantially higher connectivity than the remaining members, indicating their potential importance in preserving microtubule cytoskeleton integrity. Additionally, the network was enriched with many proteins related to microtubule nucleation, spindle formation, and cell division, including proteins associated with the GCP complex centered on GCP3. The interaction network also included γ-tubulin proteins like *TUBG1* and *TUBG2*, as well as functional proteins involved in microtubule stability regulation, such as NEDD1 and emb1427. Based on *A. thaliana* orthologs, the predicted interaction network suggested potential functional associations among *PbTUA*, *PbTUB*, and several microtubule-associated proteins. Overall, this network suggested that these proteins might collectively participate in the assembly, dynamic regulation, and functional maintenance of the plant cell cytoskeleton.

## 4. Discussion

In the genetic breeding of *P. bournei*, polyploids can provide abundant genetic diversity for this species, making it promising to breed new varieties with stronger stress resistance, higher wood yield and better wood quality. Multiple factors determine the outcome of colchicine-mediated polyploidy induction, such as colchicine dosage, treatment length, explant origin, and the endogenous sensitivity of the studied species. Extended colchicine exposure or excessively high concentrations often caused morphological abnormalities and reduced plant vigor [40]. Gupta, et al. [41] investigated the effects of different colchicine concentrations (0.00625–0.1%) on *Nigella sativa* seeds. A separate study assessed colchicine mutagenesis effects on *Vigna unguiculata* var. *sesquipedalis*. The study demonstrated that soaking in 0.09% colchicine for 4 h was the most effective treatment combination [42]. Accordingly, colchicine-treated seeds serve as a feasible approach for polyploid induction at present. In our study, germinating *P. bournei* seeds were treated with 1.0% colchicine for 24, 48 and 72 h to induce polyploid production.

Microtubules regulate plant cell division through cycles of assembly and disassembly throughout the cell cycle. Colchicine bound β-tubulin by interacting with residues 1–46 of the 16-kDa N-terminal region together with residues 214–241 of the 35-kDa C-terminal region, thereby forming a colchicine–tubulin complex [43]. Such binding suppressed microtubule polymerization and blocked kinesin family proteins (e.g., KINID1a and Kin7) from engaging in spindle construction and chromosome separation [6]. Microtubule depolymerization into soluble αβ-tubulin dimers triggered both qualitative and quantitative modifications. These alterations involved post-translational modifications, including tyrosination and ubiquitination, as well as interactions with microtubule-associated proteins such as stathmin and CLIP-170 [44]. Additionally, accurate mitosis was highly dependent on normal microtubule dynamics and the mechanical integrity of the mitotic spindle. Both excessive stabilization and destabilization of microtubules could compromise mitotic accuracy. Defects in chromosome alignment or segregation frequently gave rise to aneuploid cells. Colchicine suppressed cytokinin-related gene expression, which might reduce cellular activity, promote a shift toward anaerobic respiration, and eventually trigger late-stage apoptosis.

Substantial variation in tubulin gene copy number among plant species is mainly linked to gene duplication events and lineage-specific evolutionary trajectories [45]. The conservation and divergence of tubulin gene expansion are important for understanding potential differences in growth characteristics between *P. bournei* and other perennial woody plants. Gene duplication and subsequent divergence influence the evolution of new functions. In this study, six TUA genes and ten TUB genes were identified in *P. bournei*. The number of *PbTUA* genes is consistent with that in *A. thaliana* [46] and maize [47], but less than in hexaploid wheat (*n* = 15) [48], *Populus* (*n* = 8) [49], and *Salix arbutifolia* (*n* = 8) [20]; the number of *PbTUB* genes is greater than in *A. thaliana* (*n* = 9), maize (*n* = 8), and rice (*n* = 8), but less than in wheat (*n* = 28), *Populus*, and *Salix arbutifolia* (*n* = 20). Major modes of gene duplication cover whole-genome or segmental duplication, tandem duplication, transposon-mediated duplication, and retrotransposon duplication [50]. In contrast to the whole-genome duplication events that accompanied the evolution of hexaploid wheat, collinearity analysis in *P. bournei* revealed only segmental duplication events among *PbTUA* and *PbTUB* genes, which may partly explain why the number of *PbTUB* genes is much lower than that in hexaploid wheat. This observation is similar to that in *Populus deltoides*, where 16 tubulin paralogs have been identified; 15 of them originated from segmental duplication. Compared with many other eukaryotic genomes, plant genomes often show higher rates of structural variation, resulting in greater genomic diversity. Future studies should further elucidate the evolutionary relationships and functional diversification of tubulin genes in *P. bournei*.

The intron number, length, and genomic distribution of tubulin genes differ considerably among evolutionary lineages [51]. Introns play important roles in alternative splicing and gene expression regulation [52]. Alpha-tubulin exhibit distinct intron patterns: Class I members generally possess 31/32, 109/110, and 233/234 introns, while Class II members usually contain four introns: 38, 109, 176/177 [21]. *PtTUA* contained three and four introns. *PtTUB* genes had two conserved introns in coding sequences, plus an extra 5′-UTR intron in Class I members. In *P. bournei*, the four Class I *PbTUA* genes contained two to three introns, whereas the two Class II *PbTUA* genes, *PbTUA1* and *PbTUA4*, each contained four introns. A second intron position was found to be conserved among all *PtTUAs* in *Populus*. However, this pattern was not observed in *P. deltoides*, and no conserved intron position was detected across all *PbTUA* genes in the present study [21]. Although the intron distribution pattern of β-tubulin was more strictly conserved in angiosperms, conserved intron positions were not observed across all *PbTUB* genes. The 863 bp intron residing in the coding sequence of rice *Ostub16* was indispensable for robust transcription of downstream genes and contributed to gene expression enhancement and modulation [53]. Nevertheless, it remained elusive whether such intron-driven transcriptional enhancement was evolutionarily conserved for TUB homologs across other plants.

Tubulin genes may be regulated by developmental stages, tissue or organ specificities, and various internal and external stimuli. Tubulin genes exhibit different expression levels at specific developmental stages [33]. In *A. thaliana*, *AtTUB1* and *AtTUB5* exhibited preferential expression in roots and leaves, respectively, whereas *AtTUB9* was highly expressed in pollen. Most tubulin genes in species such as *Salix psammophila* [54] and *Camelina sativa* [55] showed elevated expression in roots and stems. However, *P. deltoides* displayed a distinct tissue-expression feature: five of the *PdTubulins* were highly expressed in roots, 12 in stems, and eight in leaves. In *P. bournei*, most *PbTUA* and *PbTUB* genes showed expression patterns similar to those reported in these species. These tissue-specific expression profiles suggested that distinct tubulin isoforms were likely to fulfil specialized roles in different plant tissues and cell types. Notably, *PbTUA4* exhibited a pronounced expression preference in leaf tissues. The relatively high transcript abundance of *PbTUA4* in leaves may meet the substantial demand for microtubule subunits to sustain active cell proliferation and morphological differentiation during leaf development. Nevertheless, whether the elevated expression of *PbTUA4* can directly trigger cell division, enhance cambial activity, or facilitate secondary growth in *P. bournei* remains unconfirmed. Although the functions of the *PbTUA* and *PbTUB* genes have not been experimentally verified, evidence from other plant species provides useful references. For example, a G-to-A substitution within the maize α-tubulin 4 gene is the main cause of the drought-overly-sensitive 1 (*ZmDos1*) pathogenic mutation, resulting in an amino acid substitution at position 196 [56]. In this study, most *PbTUA* and *PbTUB* genes were repressed under colchicine exposure, whereas *PbTUB6* escaped such transcriptional suppression and exhibited a distinct expression profile. Notably, subcellular localization analysis predicted that *PbTUB6* was primarily localized in the cytoplasm, whereas other *PbTUB* genes were predicted to localize to the nucleus. Besides, sequence polymorphism especially within the C-terminal acidic tail and colchicine-binding pocket may alter colchicine-binding affinity of *PbTUB6*, yet this hypothesis requires further experimental validation. Taken together, these observations suggested that colchicine exposure broadly influenced tubulin gene expression during the early response of *P. bournei* seedlings. It should be noted that our conclusions are bioinformatics-driven, and further experimental validations are required to confirm the biological roles of these candidate tubulin genes. However, further experimental studies are required to determine the regulatory functions and molecular mechanisms of specific *PbTUA* and *PbTUB* genes.

## 5. Conclusions

In this study, six *PbTUA* genes and ten *PbTUB* genes were identified based on the whole genome of *P. bournei*. The 16 tubulin genes were divided into two TUA subfamilies and five TUB subfamilies, and they were unevenly distributed across seven chromosomes. *PbTUA* and *PbTUB* showed a closer phylogenetic relationship to dicotyledonous woody plants and had a total of 30 collinear gene pairs with *P. trichocarpa*. Tissue expression analysis revealed that the majority of tubulin genes displayed relatively high transcript abundance in roots and stems. After colchicine treatment with different time gradients, nearly all *PbTUA* and *PbTUB* genes were downregulated, whereas several members were upregulated to different extents at 72 h. In particular, *PbTUB6* exhibited up-regulation under colchicine treatment, and cytoplasmic localization. Collectively, these observations offered theoretical support for understanding the functional roles of *PbTUA* and *PbTUB* genes and supported further exploration of colchicine-triggered polyploidization mechanisms in *P. bournei*.

## Figures and Tables

**Table 1:** Physicochemical characteristics and predicted subcellular localization of *PbTUA* and *PbTUB* genes of *P. bournei*.

Gene Name	Gene ID	Number of Amino Acid	Molecular Weight/Da	pI	Instability Index	GRAVY	Subcellular Status
*PbTUA1*	OF30059	414	45,809.8	5.01	35.31	−0.16	Cytoskeleton
*PbTUA2*	OF20834	451	49,597.92	4.93	36.11	−0.194	Cytoplasm
*PbTUA3*	OF04782	451	49,607.92	4.96	35.85	−0.192	Cytoplasm
*PbTUA4*	OF04549	450	49,771.31	4.92	36.61	−0.145	Cytoplasm
*PbTUA5*	OF25888	450	49,607.96	4.97	37.46	−0.2	Cytoplasm
*PbTUA6*	OF10968	451	49,597.92	4.93	36.11	−0.194	Cytoplasm
*PbTUB1*	OF26575	447	50,267.55	4.73	38.27	−0.368	Nucleus
*PbTUB2*	OF11660	446	50,194.51	4.75	36.62	−0.373	Nucleus
*PbTUB3*	OF03531	522	58,841.7	4.96	42.07	−0.604	Nucleus
*PbTUB4*	OF24043	449	50,362.63	4.75	36.26	−0.382	Nucleus
*PbTUB5*	OF23459	430	48,269.44	4.76	35.38	−0.339	Nucleus
*PbTUB6*	OF01600	445	50,181.48	4.73	39.88	−0.385	Cytoplasm
*PbTUB7*	OF05182	449	50,338.61	4.76	37.02	−0.359	Nucleus
*PbTUB8*	OF29906	474	53,351.5	4.79	41.41	−0.261	Nucleus
*PbTUB9*	OF17418	437	49,252.41	4.7	37.52	−0.394	Nucleus
*PbTUB10*	OF15890	445	50,156.45	4.76	38.42	−0.375	Nucleus

## Data Availability

Statistical analyses are included in the Supplementary Materials. Raw transcriptome data have been deposited in the NCBI Sequence Read Archive (SRA) under the BioProject accession PRJNA1496853 (https://www.ncbi.nlm.nih.gov/bioproject/PRJNA1496853, accessed on 18 July 2026), with the four datasets used in this study corresponding to SRA accessions SRR39685744 (MCK0), SRR39685736 (TR10_24), SRR39685735 (TR10_48), and SRR39685734 (TR10_72). All data will be made publicly available upon publication of this article.

## Supplementary Materials

The following supporting information can be downloaded at https://www.mdpi.com/article/10.3390/biology15171456/s1, Figure S1: Observation of the trees in *P. bournei* for material collection (A) Trees in *P. bournei*; (B) Panicle inflorescence of *P. bournei*; (C) Morphology of a single flower of *P. bournei*; Table S1: Protein sequences used in this study; Table S2: Expression data of *PbTUA* and *PbTUB* genes; Table S3: Ka/Ks analysis in the tubulin gene family of *P. bournei*; Table S4: Top 20 enriched KEGG pathways of DEGs.

## Abbreviations

The following abbreviations are used in this manuscript:

*P. bournei*	*Phoebe bournei*
*A. thaliana*	*Arabidopsis thaliana*
GFF	General Feature Format
FPKM	Fragments Per Kilobase of transcript per Million mapped fragments
GMQE	Global Model Quality Estimate
GO	Gene Ontology
KEGG	Kyoto Encyclopedia of Genes and Genomes
DEGs	Differentially expressed genes
BP	Biological process
MF	Molecular function
CC	Cellular component
