# Supplementary material for: Genome-Wide Identification and Colchicine-Responsive Expression Profiling of the Tubulins (TUA and TUB) Gene Family in Phoebe bournei
Source: Biology (Basel). 2026 Aug 26;15(17):1456. doi: 10.3390/biology15171456 (PMC13565121; doi:10.3390/biology15171456)
Supplement: Supplementary file 1 [file biology-15-01456-s001.zip › Supplementary Material/Supplementary Material.pdf]

Supplementary Material

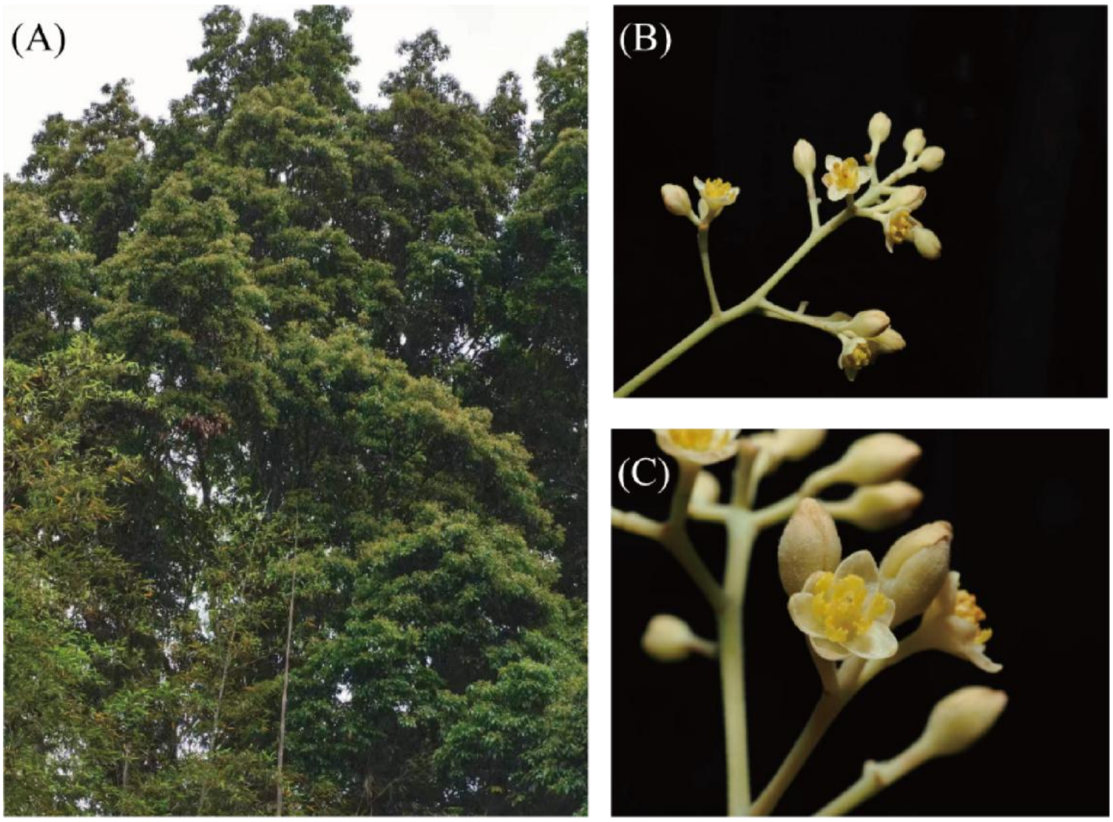

Figure S1. Observation of the trees in *P. bournei* for material collection. (**A**) Trees in *P. bournei*; (**B**) Panicle inflorescence of *P. bournei*; (**C**) Morphology of a single flower of *P. bournei*.

Table S3. Ka/Ks analysis in the tubulin gene family of *P. bournei*

Gene Name	Gene ID	Gene Name	Gene ID	Ka	Ks	Ka/Ks	Duplication Type
PbTUA2	OF20834	PbTUA3	OF04782	0.006838	1.38437	0.00493918	Segmental duplication
PbTUA2	OF20834	PbTUA6	OF10968	0.006845	1.215568	0.00563149	Segmental duplication
PbTUA3	OF04782	PbTUA6	OF10968	0.003901	0.766923	0.00508597	Segmental duplication
PbTUB2	OF11660	PbTUB5	OF23459	0.014043	0.62548	0.02245129	Segmental duplication
PbTUB6	OF01600	PbTUB8	OF29906	0.013717	0.985694	0.01391599	Segmental duplication
PbTUB4	OF24043	PbTUB7	OF05182	0.014061	0.993208	0.01415717	Segmental duplication
PbTUB6	OF01600	PbTUB8	OF29906	0.013717	0.985694	0.01391599	Segmental duplication
PbTUB4	OF24043	PbTUB9	OF17418	0.012919	0.620975	0.02080359	Segmental duplication
